# Subcutaneous and Mediastinal Emphysema Followed by Group A Beta-Hemolytic Streptococci Mediastinitis. A Complicated Course after Adenotonsillectomy: Case Report

**DOI:** 10.3390/diagnostics9010011

**Published:** 2019-01-15

**Authors:** Anne Duvekot, Gwen van Heesch, Laura Veder

**Affiliations:** 1 Department of Otorhinolaryngology and head and neck surgery, Erasmus Medical Center, Sophia Children’s Hospital, 3015 GD Rotterdam, The Netherlands; l.veder@erasmusmc.nl; 2 Department of Pediatrics, Pediatric Intensive Care Unit, Erasmus Medical Center, Sophia Children’s Hospital, 3015 GD Rotterdam, The Netherlands; g.vanheesch@erasmusmc.nl

**Keywords:** tonsillectomy, subcutaneous emphysema, mediastinal emphysema, mediastinitis, complication, bacteremia

## Abstract

Tonsillectomy is a commonly performed surgery in the daily practice of an otorhinolaryngologist. For patients as well as health professionals, the best known complication is post-operative bleeding. Among the less noted, but potentially life-threatening, complications are the development of subcutaneous emphysema and the presence of bacteremia due to group A hemolytic streptococci. In this report, we describe a severely complicated clinical course after an uncomplicated adenotonsillectomy in a young boy. Increased awareness of relatively unknown complications after adenotonsillectomy amongst surgeons, pediatricians and anesthesiologists is desirable to facilitate rapid diagnosis and adequate treatment in order to prevent life-threatening situations.

## 1. Introduction

Adenotonsillectomy is one of the most frequently performed surgical procedures in the daily practice of an otorhinolaryngologist, especially in children. Nevertheless, the operation is associated with several severe pre- and post-operative complications. According to literature, primary or secondary bleeding, pain and temporary taste disorders are the most common complications [1,2,3,4,5]. In the Netherlands, according to the national guideline, potential postoperative bleeding and taste disorders are obliged to be mentioned by the otorhinolaryngologist during the pre-operative intake [2,3,6,7]. Other less frequent complications include damage to teeth, otalgia, lingual edema, injury to the glossopharyngeal nerve and injury to the carotid artery [4]. Infectious complications are not frequently reported but can be serious in case of bacteremia [8]. Furthermore, a known but rare and potentially life-threatening complication is the development of (cervico)facial subcutaneous and/or mediastinal emphysema [9,10,11,12,13].

In this case report, we describe the case of a young boy with cervicofacial subcutaneous and mediastinal emphysema after adenotonsillectomy followed by a serious systemic infection caused by group A beta-hemolytic streptococci. We will summarize the current literature on cervicofacial subcutaneous and/or mediastinal emphysema and infectious complications after adenotonsillectomy.

## 2. Case Report

A 22-month-old boy with Down syndrome was admitted to our department for elective adenotonsillectomy because of sleep apnea due to adenoidal and tonsillar hypertrophy. Despite conservative treatment for the previous five months with nasal rinsing and intranasal steroid spray, his symptoms had deteriorated and he was indicated for surgery with post-operative observation overnight. Except for the presence of habitual belching, his medical history revealed no other abnormalities.

After a nontraumatic and uneventful orotracheal intubation, guillotine adenotonsillectomy was performed under general anesthesia. Hemostasis was achieved by dry gauze compress on both sides, no bipolar cautery was necessary. Both tonsils were easily removed without remarkable adhesions and there was no excessive bleeding during or after the procedure. Our patient was monitored in the recovery room and discharged to the ward after 40 min. 

The next morning during visitation rounds, the patient’s dismissal home was postponed due to inadequate fluid intake. The physical examination and vital signs showed no aberrant findings. In the afternoon the patient was reassessed because of a swelling on the right side of his face. His vital signs were normal and there was no fever or difficulty breathing. Further physical examination showed facial swelling on the right side and crepitus was felt during palpation. There were no signs of cellulitis. Oral examination of the tonsil bed revealed normal wound healing without obvious mucosal tears. Ultrasound confirmed the presence of subcutaneous emphysema. 

The progression of the emphysema quickly resulted in signs of an obstructed airway with a saturation of 81% SpO2 and the use of accessory muscles of respiration. The patient was quickly transferred to the pediatric intensive care unit where he was intubated. Shortly after successful intubation, the patient went in cardiac arrest and 2 min of cardiopulmonary resuscitation was performed. Bedside evaluation arrest ruled out pneumothorax, cardiac tamponade, hypoxia or airway obstruction and hypovolemia. Cardiac ultasonography showed a diminished ventricular function.

**Figure A1:**
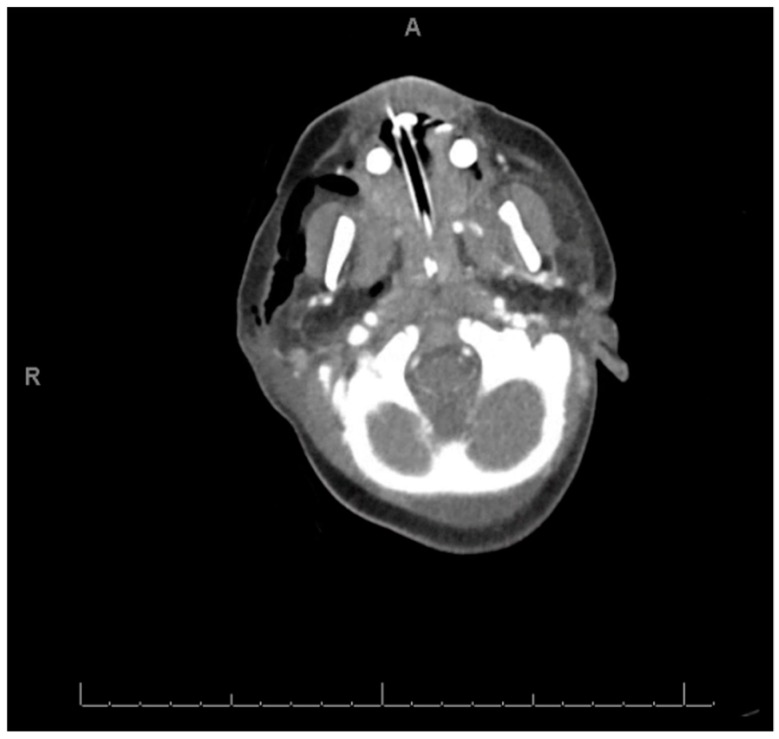
Computed tomography scan of our patient taken two days after the adenotonsillectomy. The image shows infiltrative soft-tissue and subfascial emphysema in the facial region particularly on the right side.

Blood tests showed white blood cell count of 0.78 × 10^3^e/uL and a C-reactive protein of 142 mg/L. Blood cultures were positive with group A beta-hemolytic streptococci. A computed tomography scan (Appendix Figure A1) revealed mediastinal infectious infiltration besides subcutaneous emphysema. In retrospect, the cause of the cardiopulmonary arrest turned out to be due to sepsis/mediastinitis.

Our patient was treated with broad spectrum antibiotics, inotropes and intravenous immunoglobulin. The further course was uneventful. The subcutaneous emphysema resolved over the following days and the patient could be extubated 6 days after admission to the PICU. Finally, 15 days after surgery he was discharged home. Fortunately, follow-up consultations showed no neurological residual symptoms.

## 3. Discussion

Subcutaneous emphysema has been described in the literature as a complication following tonsillectomy. The seriousness of emphysema varies and includes bilateral cases, pneumomediastinum, pneumothorax and even pneumoperitoneum.

Massive emphysema can compress the trachea, especially in young children, since their tracheal rings are soft. Due to the connection between the parapharyngeal and retropharyngeal space, emphysema can subsequently spread to the mediastinum, causing a pneumomediastinum and even pneumothorax, with major respiratory consequences [14,15]. The typical clinical finding by physical examination is crepitus on palpation. Other diagnoses such as hematoma, allergic reaction or necrotizing fasciitis have to be excluded. 

The latter is especially important to distinguish, since rapid treatment with intravenous antibiotics and surgical debridement can be curative. Local tenderness and erythema with variant vital signs are warning signs of infection [16].

It has been suggested that patients with severe adhesions of the tonsil to the superior constrictor muscle, for example after recurrent tonsillitis or peritonsillar abscess, are more prone to develop postoperative subcutaneous emphysema. Due to the adhesions, the dissection of the tonsils from the tonsillar bed can be difficult, increasing the risk of injury to the tonsillar fossa [12]. Accordingly, assuming that removing pediatric tonsils is easier, we found only three cases of pediatric patients with subcutaneous emphysema since 2000, with ages of 6, 7 and 11 years [15,17,18].

Although the exact pathophysiological mechanism is still unknown, “ascending” and “descending” routes are described [9,17,19].

In the ascending mechanism, a rupture occurs anywhere along the tracheobronchial tree from a pre-existing abnormality such as an alveolar bleb or laryngocele or from an acquired defect from traumatic intubation. Following excessive positive pressure ventilation or because of increased intrathoracic pressure, the pre-existing defect can rupture causing the entry of air into the peritracheal or perialveolar tissue. In the case of an alveolar rupture, air can pass through the perivascular sheaths into the mediastinum. Through the subfascial tissue, air can ‘ascend’ to the subcutaneous tissues of the neck and facial area [17].

In the descending pathway, subcutaneous air infiltration into the pharyngeal space is caused by deep dissection into the tonsil fossa, causing a breach of the superior constrictor muscle and the underlying fascia. Increased intrathoracic pressure (coughing, vomiting, physical exertion, straining and/or manual positive pressure mask ventilation) allows air to migrate through the defect. 

Since the subcutaneous emphysema was initially limited to our patient’s face and there was a nontraumatic intubation, we consider the ‘descending’ pathway to be more likely.

The habitual belching also supports the presence of the descending mechanism. 

In order to prevent the occurrence of subcutaneous emphysema, it is important to perform careful surgery. In our patient, we performed a tonsillectomy using the Guillotine technique, according to the Dutch guideline. The aim, as in all techniques, is to remove the tonsils completely with minimal hemorrhage and minimal postoperative complications [20]. However, to date there is no international consensus on which technique is best to perform a tonsillectomy. In the case reports describing the development of subcutaneous emphysema, different techniques were used (i.e., electro-dissection with bipolar scissors, tonsil dissector, Ultracision Harmonic scalpel and cold steel elevators). Conclusions about the used technique and its role in the development of subcutaneous emphysema cannot be made. A tonsillotomy (intracapsular, partial or subtotal tonsillectomy), in which the tonsil parenchyma is removed without damage to the tonsillar fossa and the superior constrictor muscle, might minimalize the risk of postoperative subcutaneous emphysema [20,21]. Furthermore, if possible, patients can be instructed to avoid activities that increase intrathoracic pressure. Unfortunately, our patient could not repress the belching.

The treatment of subcutaneous emphysema is mostly conservative but includes broad-spectrum antibiotics to prevent infection from the oral cavity. In case of an evident mucosal rupture, reparation of the damage is recommended. Medication to suppress increasing intrathoracic pressure, such as anti-emetics, codeine or stool softeners can be prescribed [9]. In most cases the subcutaneous emphysema resolves spontaneously and no further treatment is necessary. Of the 43 cases described in a review by Saravakos, three patients needed re-intubation, one patient required temporary tracheostomy to secure their airway and three patients underwent thoracotomy for air drainage out of the mediastinum [9,10,18,22,23,24,25].

Our patient was intubated because of progressive, airway-obstructing emphysema, but despite effective airway management he deteriorated due to a systemic infectious disease. 

Bacteremia after tonsillectomy has also been described and is relatively innocent in most cases. According to the literature, transient bacteremia occurs in 22% to 70% of all post-tonsillectomy patients and generally ceases within 20 to 40 min in healthy patients [26]. The most commonly responsible micro-organism is the Haemophilus influenza. Although group A beta-hemolytic streptococci are frequently found in local tonsillar oropharyngeal cultures, they are rare in blood cultures and we found only two previous cases describing the presence of a severe systemic group A streptococcal infection in otherwise healthy patients after tonsillectomy. In the first case, a four-year-old girl developed a severe streptococcal septic shock syndrome the night after surgery. In the second case, a four-year-old boy revealed invasive streptococcal infection symptoms 5 days after surgery. He died 4 days after re-admission due to a ruptured mycotic aneurysm of the ascending aorta and hemorrhagic pericarditis caused by group A streptococcal sepsis [8].

Invasive infections with group-A-streptococci can be devastating due to endotoxin release resulting in a septic shock syndrome. The overall mortality of such streptococcal septic shock syndrome is around 50% [27].

Although rare, serious infectious complications after tonsillectomy can occur and should be kept in mind. Prompt recognition and treatment are necessary. Authors should discuss the results and how they can be interpreted in the context of previous studies and of the working hypotheses. The findings and their implications should be discussed in the broadest context possible. Future research directions may also be highlighted.

## 4. Conclusions

We described a severely complicated clinical course after an uncomplicated adenotonsillectomy. Since the development of subcutaneous emphysema as well as the presence of a group A hemolytic streptococci bacteremia after tonsillectomy are both rare but life-threatening complications, we should all be aware of their (co-)existence. Intensive co-operation by surgeons, pediatricians and anesthesiologists is necessary for fast and adequate diagnosis and treatment to prevent further damage.
